# Investigation on the site of coronal deformities in Hallux valgus

**DOI:** 10.1038/s41598-023-28469-4

**Published:** 2023-02-01

**Authors:** Rachel Xiaoyu Wei, Violet Man-chi Ko, Elvis Chun-sing Chui, Bruma Sai-chuen Fu, Vivian Wing-yin Hung, Patrick Shu-hang Yung, Samuel Ka-kin Ling

**Affiliations:** 1 Department of Orthopaedics and Traumatology, Faculty of Medicine, The Chinese University of Hong Kong (CUHK), Prince of Wales Hospitale, Lui Che Woo Clinical Science Building, 5/F, Shatin, Hong Kong SAR China; 2 Department of Orthopaedics, Southwest Hospital, Third Military Medical University (Army Medical University), Chongqing, China

**Keywords:** Preclinical research, Translational research, Anatomy, Surgery

## Abstract

Hallux valgus (HV) is a common foot deformity that is more prevalent in females, characterised by abnormal adduction of the first metatarsal (MT) and valgus deviation of the phalanx on the transverse plane. Increasing evidence indicates that HV is more than a 2D deformity but a 3D one with rotational malalignment. Pronation deformity is seen during clinical examination for HV patients, but the exact origin of this rotational deformity is still unknown. Some attribute it to first tarsometatarsal (TMT) joint rotation, while others attribute it to intra-metatarsal bony torsion. In addition, the correlation between the rotational and transverse plane deformity is inconclusive. Identifying the origin of the rotational deformity will help surgeons choose the optimal surgical procedure while also enhancing our understanding of the pathophysiology of HV. This study aims to (1) develop an objective method for measuring the first MT torsion and first TMT joint rotation; (2) investigate the exact location of the coronal deformity in HV; (3) investigate the relationship between the severity of deformity on the transverse and coronal planes as well as the correlation between deformity severity and foot function/symptoms in HV. Age-matched females with and without HV were recruited at the Foot and Ankle Clinic of the Department of Orthopaedics and Traumatology. Computed tomography was conducted for all subjects with additional weight-bearing dorsal-plantar X-ray examination for HV subjects. Demographic information of all subjects was recorded, with symptoms and functions related to HV evaluated. The intra-class correlation was used to explore the relationship between deformities on different planes and the deformity severity and functional outcomes, respectively. An Independent *t*-test was used to compare joint rotation and bone torsion degrees. TMT joint rotation is significantly correlated with foot function. HV patients had more TMT joint rotation but not MT torsion compared to normal controls. No relationship was found between the coronal rotation and the 1,2-intermetatarsal angle (IMA) or Hallux valgus angle (HVA) on the transverse plane. Our results indicate that coronal deformities in HV may originate from TMT joint rotation. In addition, the severity of the TMT joint coronal rotation correlates with worse foot function; thus, multi-plane assessment and examination will be necessary for more precise surgical correction.

## Introduction

Hallux valgus (HV) is a common forefoot deformity, with a prevalence of 23% in 18–65 years adults and even higher in the elderly population aged over 65 years^1^. Patients with HV suffer from pain due to inflammation and deformity-related problems such as difficulty fitting in shoes and pressure ulcerations^2^. Conservative treatment may contribute to pain alleviation, but its power for deformity correction is limited^3^. A previous review found surgery was more beneficial than conservative interventions, especially in moderate to severe deformities^4^.

A wide range of surgical procedures has been reported to show promising results. Currently, 1,2 intermetatarsal angle (IMA) and hallux valgus angle (HVA) are the mainstream radiological measurements used to assess HV realignment. The operations aim to reduce 1,2 IMA to less than 9° and HVA to less than 15°^5^. The transverse measurements observed on the plain radiographs are important; however, solely looking at the transverse radiological parameters fails to account for the coronal-plane deformity, as increasing evidence appears to support multiplanar deformities in HV^6^.

To precisely correct all the existing multiplanar deformities, it is essential to accurately determine the deformity site and calculate how much correction is required. Therefore, considering the coronal component is necessary during pre-operative planning to fully correct the deformity. However, the location of the coronal deformity in HV remains unclear as most studies report a gross rotational deformity without specification of where it rotates. It remains unclear whether internal bony torsion within the first metatarsal bone (MT) or joint rotation at the first tarsometatarsal (TMT) joint is the main contributor to the overall pronation.

In addition, function and symptoms are not always directly correlated with plain dorsal-plantar radiographic deformity severity in the transverse plane^7^, indicating that the entire clinical phenomenon is still not fully understood. It is plausible to suppose that some important factors may be overlooked in our current traditional clinical examination. Based on these considerations, we believe it is crucial to understand whether the coronal deformity in HV is dependent or independent of the transverse deformities and whether the coronal malalignment is associated with foot function. Unravelling the complex demonstration of HV will allow for more precise patient-specific treatment in the future.

The study aims to (1) develop an objective method for measuring the first MT torsion and first TMT joint rotation; (2) investigate the exact location of the coronal deformity in HV; (3) investigate the relationship between the deformity severity on the transverse and coronal planes as well as the correlation between deformity severity and foot function/symptoms in HV.

## Methods

### Ethics approval

The current study was approved by the Joint Chinese University of Hong Kong—New-Territories East Cluster Clinical Research Ethics Committee. Written informed consent was obtained from all the subjects, and the experiments were carried out per the Declaration of Helsinki.

### Sample size estimation

The sample size estimation is based on the results from a previous study that investigated the first MT torsion between HV and non-HV groups. The effect size Cohen’s *d* was calculated by each group’s mean and standard deviation (HV group: 17.6 ± 7.7, n = 27; non-HV group: 4.7 ± 4.0, n = 27). With setting alpha = 0.05, 1-beta = 0.95, effect size = 2.10, the sample size was calculated using G*Power version 3.1.9.6. According to the result, a minimum of 16 cases was required, with 8 in each group.

### Inclusion and exclusion criteria of subject recruitment

The subjects for the HV group were all recruited from the Foot and Ankle Clinic at the Prince of Wales Hospital. To be included in the current study, the patients had to be (1) aged from 18 to 75 years; (2) females; (3) diagnosed clinically (by surgeons from the Foot and Ankle team) and radiographically (HVA > 15° or IMA > 9° on the weight-bearing anteroposterior X-ray view). The patients who had (1) recurrence of Hallux valgus; (2) any unstable medical complications; (3) any trauma history known to possibly affect the anatomical structure or morphology of the foot such as fracture, rheumatoid arthritis, congenital malformation; (4) any history of operation at the involved foot; (5) pregnancy were excluded.

The subjects for the control group were patients from the same outpatient clinic and the convenient samples (e.g. visitors or colleagues from the Department of Orthopaedics and Traumatology). They must satisfy the following criteria: (1) aged from 18 to 75 years; (2) female; (3) at least one asymptomatic foot that does not suffer pain or deformity; (4) have no trauma history known to possibly affect the anatomical structure or morphology of foot such as fracture, rheumatoid arthritis, congenital malformation; (5) have no history of operation at the involved foot; (5) pregnancy or other conditions which are not appropriate for CT scanning.

### Experiment methods

#### Foot-related function evaluation

The patients who satisfied the inclusion criteria for the HV group were asked to complete the Foot and Ankle Outcome Score function evaluation (FAOS)^8^. The FAOS is a patient-reported outcome sore that includes 42 items split into five categories to assess a patients’ pain (9 items), (2) other symptoms (7 items), activities of daily living (ADL) (17 items), sporting ability (5 items) as well as the quality of life (QoL) (4 items) related to the foot according to individual feeling in the past week. Each item is scored by subjects based on the five-point Likert scale and then transformed to 0 (worst) -100 (best) scale.

#### CT scanning

A second-generation high-resolution peripheral quantitative computed tomography (HR-pQCT) system (XtremeCT II, Scanco Medical AG, Bassersdorf, Switzerland) was used to obtain foot images. The scanning resolution was set as 136.7 µm (voxel size, 1024 × 1024) per scan. The scanning region covered the first metatarsal bone scanning from the head of MT to the distal end of MT with a total length of 91–99 mm, depending on the size of the foot.

**Figure 1:**
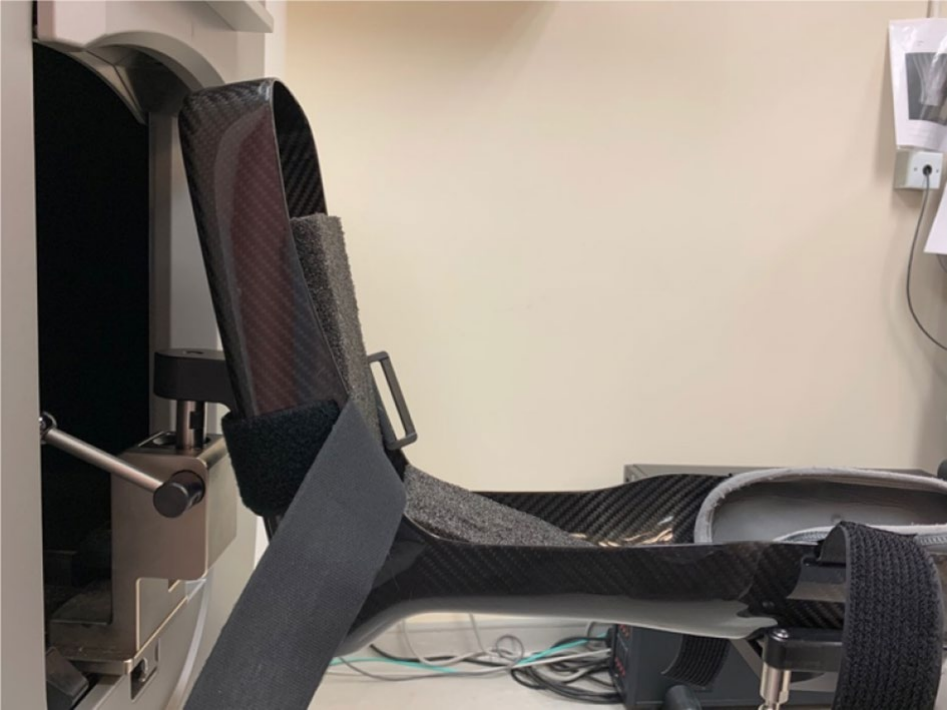
The wedge was put at the end of the splint to support the foot.

**Figure 2:**
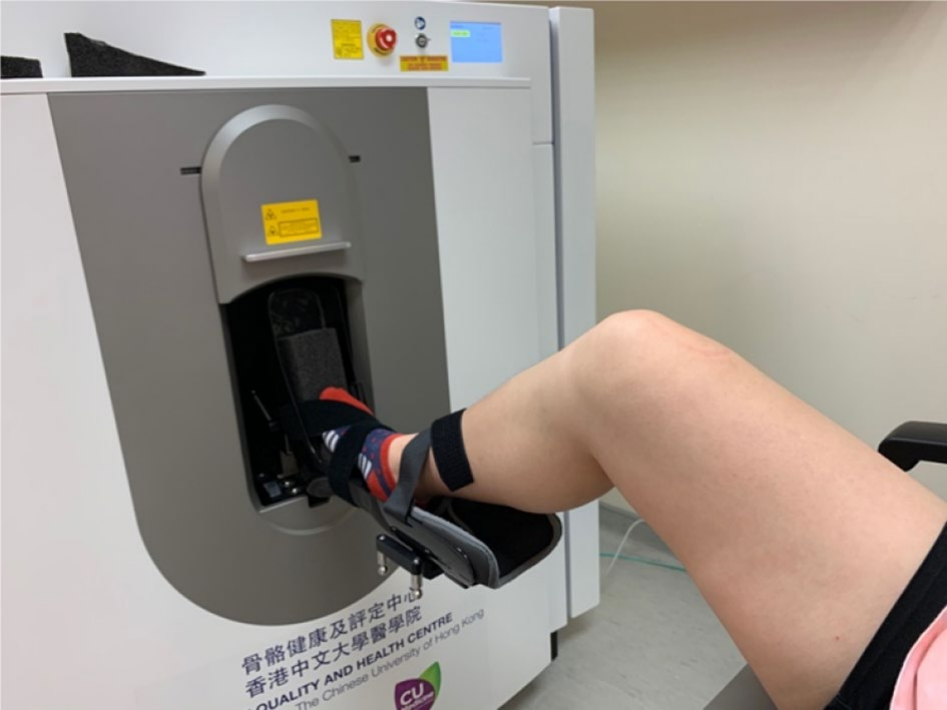
The subject’s foot was immobilised in the splint with two bandages.

At the beginning of the examination, a customised wedge was put at the end of the splint to support the foot and mimic weight-bearing (Fig. 1) while ensuring a consistent angle between foot alignment and the X-ray beam. The subject’s foot was immobilised with bandages (Fig. 2). The subjects were asked to stay as still as possible during the whole process. When starting scanning, the range of interest (ROI) was determined on the anteroposterior scout view, ranging from the proximal phalanx and ending at the navicular bone (Supplementary Fig. 1).

### Image process and data analysis

#### Coronal deformities angle measurements

All DICOM files from the CT scan were imported into the Mimics 21.0 3D image processing software (Materialise, Leuven, Belgium) for segmentation and isolating the first MT and medial cuneiform from the rest of the scan. The bone segmentation was conducted with a semi-automatic tool (Mimics Innovation Suite, Materialise, Belgium) which could automatically mark the pixels which should be bone tissue. The operator adjusted the range to ensure that pixels between bones were not included. In our study, one operator was involved in this process. The models were then exported in standard tessellation language (STL) files to the 3-Matic 11.0 (Materialise, Leuven, Belgium) for 3D reconstruction, landmarks selection and angle measurement. Three vectors were created at the first MT head (Vector A), the first MT base (Vector B) and the distal end of the medial cuneiform (Vector C), respectively. The details to create the vectors for the individual subject are presented as follows.

##### Vector A: The first MT head (distal MT reference)

**Figure 3:**
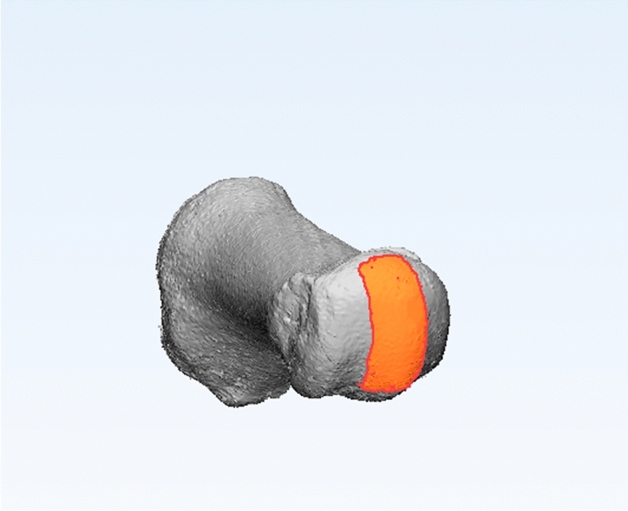
The middle one-third of the whole articular surface of the MT head was marked and extracted from the view of the distal pole.

**Figure 4:**
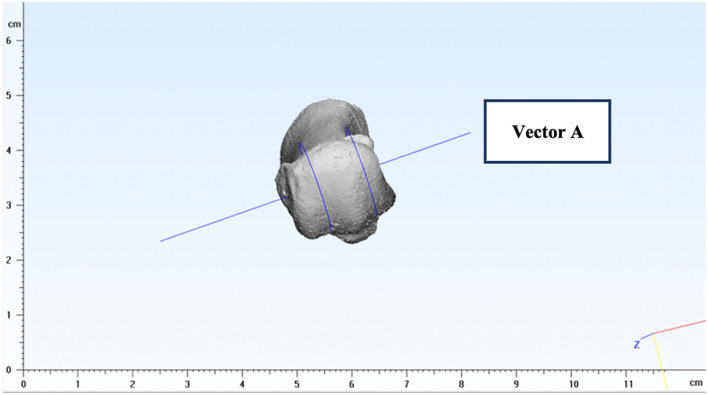
The cylinder was created by the least-square method and the axis of this fitting cylinder represented the position of the distal end of the first MT.

Creating a cylinder using the least-square method is the most common way when handling irregular surfaces. Defined as a one-axis hinge joint, the proximal segment of the first Metatarsophalangeal (MTP) joint could be analogised as a cylinder, and the articular surface could be easily divided into three parts. For the cylindrical estimation, the middle one-third of the whole articular surface of the MT head was marked and extracted from the view of the distal pole, as the regularity of the middle one-third can minimize the variation from manual selection (Fig. 3)^9–11^. The least-square method and the axis of this fitting cylinder represented the position of the distal end of the first MT (Vector A) (Fig. 4).

##### Vector B: The first MT base (proximal MT reference)

**Figure 5:**
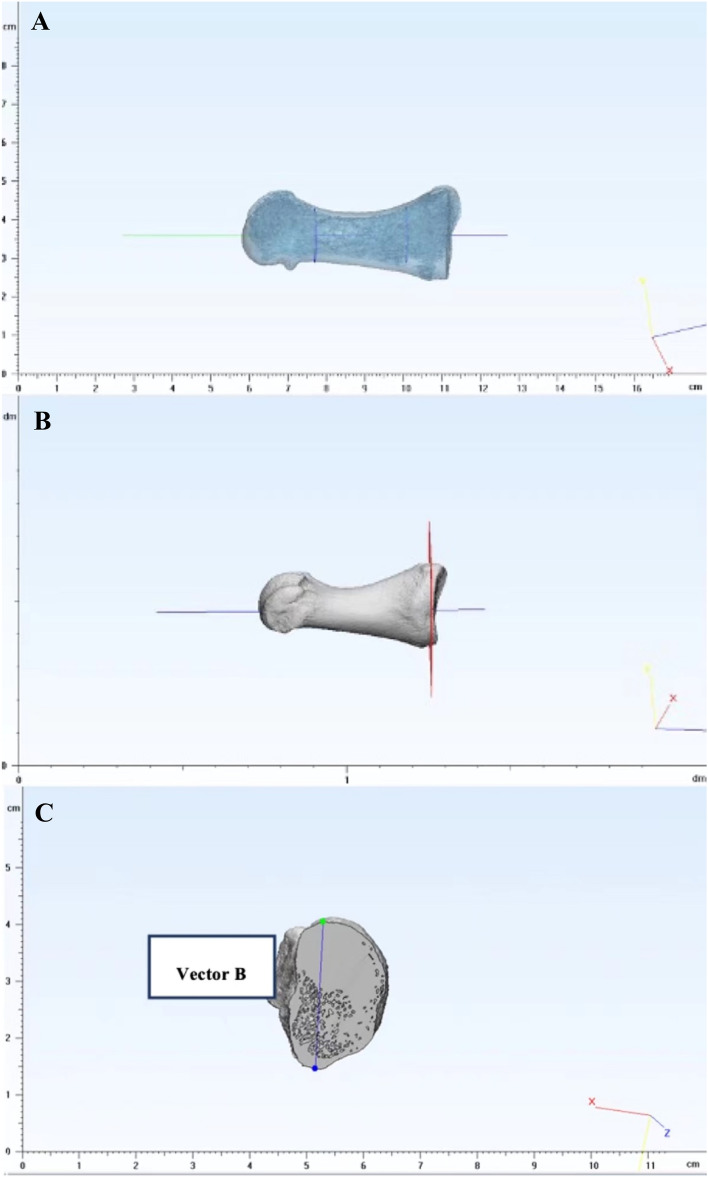
(**A**) A fitting cylinder of the diaphysis was created. (**B**) A cross-section beneath the articular surface was extracted. (**C**) Vector B was created by connecting the most superior and inferior point.

The rationale for creating Vector B, which presents the position of the first MT base, is similar to the previous studies, i.e. selecting the most superior and inferior point of the proximal MT^11–13^. To reduce the effect of erosion of the joint surface due to TMT joint arthritis when selecting anatomical landmarks^14^, we used a novel method. Firstly, the diaphyseal section of the first MT was marked, i.e. 1 cm proximal to the distal articular surface and 1 cm distal to the proximal articular surface^15^. A fitting cylinder of the marked area was created using the least-square method (Fig. 5A), the axis of which was defined as the longitudinal axis of the first MT bone. Instead of directly selecting landmarks on the articular surface, a cross-section was selected manually by moving the plane along the longitudinal axis to the location just beneath the articular surface (Fig. 5B). This step is aimed at reducing the effect of abnormal bone tissue (e.g. bone hyperplasia) at the articulation. The most superior and inferior points on this cross-section were marked, and Vector B was created by connecting the points (Fig. 5C). The reference system depends on the placement of the splint as described above, as the foot with the splint was kept static.

##### Vector C (medial cuneiform reference)

**Figure 6:**
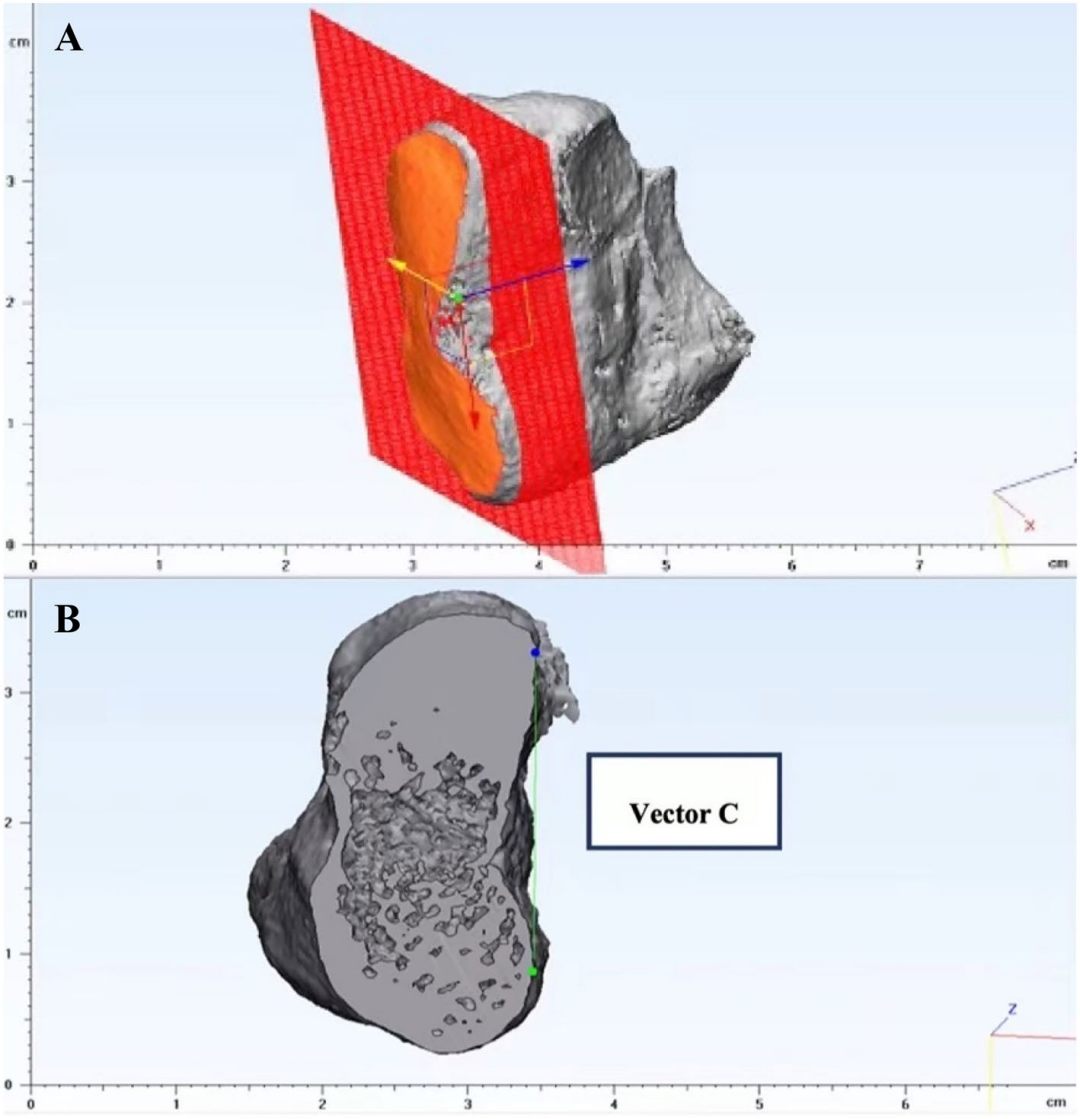
(**A**) A fitting plane was created based on the highlighted articular surface. (**B**) Vector C was created by connecting the two most lateral points on the cross-section.

As we did above, the operator manually selected a cross-section just beneath the medial cuneiform’s articular surface and then extracted. This new plane was created by making a fitting plane based on the highlighted articular surface (Fig. 6A). The two most lateral points on the cross-section were highlighted and connected to create Vector C (Fig. 6B). Like the reference system above, the reference system to define the points was also based on the splint.

##### Angle definition

**Figure 7:**
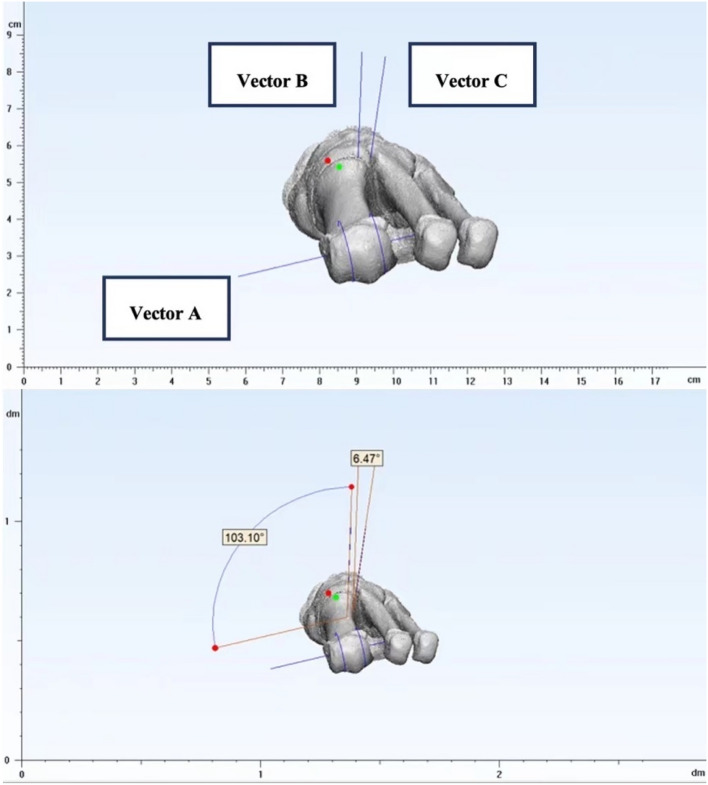
The definition of first MT torsion angle and TMT joint rotation angle.

For each individual, the internal torsion angle of the first MT was defined as the angle between Vector A (distal MT reference) and B (proximal MT reference). The rotation angle at the TMT joint was defined as the angle between Vector B (proximal MT reference) and C (medial cuneiform reference). Vector A provided the overall measurement of coronal rotation as seen clinically. With the addition of vector B (proximal first metatarsal) and vector C (medial cuneiform), the rotation region was located. If there were changes in the proximal metatarsal, the rotation was postulated to occur between Vector C and Vector B (likely the first TMTJ rotation). If the deformity occurs between vector B and Vector A, it indicates torsion within the first metatarsal bone (Fig. 7).

### Data analysis

The Shapiro–Wilk test was used to assess data normality, including TMT joint rotation angle and MT torsion angle. According to the distribution, the independent student *t*-test (normally distributed) or the Wilcoxon signed-rank test (non-normally distributed) was conducted to compare the difference between the HV group and the control group. The significance level was set as 0.05.

For patients with HV, the relationships between the coronal deformities (TMT joint rotation angle and MT torsion angle) and the transverse deformities (IMA and HVA) were studied using Pearson or Spearman correlation according to the results of the Shapiro–Wilk test. The relation between foot-related function and deformity angles was also studied using Pearson or Spearman correlation; if the statistical significance was found (*p* < 0.05), the relation was classified as “strong” (≥ 0.5), “moderate” (0.3–0.5), or “weak” (< 0.3) based on the value of correlation coefficient.

## Results

### Demographic information

The current study recruited 17 patients (23 feet) who were clinically and radiographically diagnosed with HV and 16 control subjects without HV. No difference was found between the two groups regarding gender, age, body weight and body mass index (BMI) (Table 1).

**Table 1:** Demographic information of subjects in two groups.

	HV group	Control group	*p*-value
Total number (patients/feet)	17/23	16/16	–
Age (years)	58.63 ± 13.00	51.88 ± 15.53	0.17
Gender (female/male)	19/0	16/0	–
Body weight (kg)	59.54 ± 7.42	59.30 ± 10.00	0.94
Body height (m)	1.59 ± 0.06	1.58 ± 0.06	0.91
BMI	23.80 ± 2.38	23.61 ± 3.50	0.88

### Difference in coronal deformities between HV and control

According to the results of the Shapiro–Wilk test, the first TMT joint rotation angle and the first MT torsion angle were normally distributed in two groups. Therefore, an independent *t-*test was used for further comparison.

The first TMT joint rotation angle was found as 13.09 ± 4.29° in HV and 8.16 ± 2.05° in control, with a significant difference (*p* < 0.01). The mean difference between the two groups was 4.93 ± 1.31°. The mean value (± SD) of the first MT torsion was 93.47 ± 7.00° in HV and 95.66 ± 6.18° in control. No significant difference was found (*p* = 0.36). The mean difference between the two groups was − 2.20 ± 2.37° (Table 2).

**Table 2:** Results of 1TMT joint rotation and the first MT torsion in two groups.

	HV group		Control group		Mean difference	*p*-value
	Mean (SD)	95% CI	Mean (SD)	95% CI	Mean [lower bound of 95% CI, upper bound of 95% CI]	
TMT joint rotation (°)	13.09 (4.29)	[11.32, 14.87]	8.16 (2.05)	[6.86, 9.46]	4.93 [2.27, 7.60]	< 0.01**
First MT torsion (°)	93.47 (7.00)	[90.57, 96.35]	95.66 (6.18)	[91.74, 99.59]	− 2.20 [− 7.01, 2.62]	0.36

*The correlation is significant with p-value < 0.05.

**The correlation is significant with p-value < 0.01.

### The correlation between coronal and transverse deformities

The Pearson correlation was used for the following analysis. In the HV group, the average IMA was 15.66 ± 3.12°, and HVA was 38.43 ± 7.05°. The study found no correlation between coronal and transverse deformities. Specifically, TMT joint rotation of HV patients exhibited no significant relation with IMA (*p* = 0.98, *r* = − 0.007) or HVA (*p* = 0.25, *r* = 0.248). Similarly, no significant relation was found between MT torsion and IMA (*p* = 0.88, *r* = − 0.034) or HVA (*p* = 0.22, *r* = 0.265). Increased IMA was found to be significantly associated with increased HVA (*p* < 0.01, *r* = 0.587), while no significant association was found between two coronal deformities (*p* = 0.84, *r* = 0.034).

### Correlation between deformities and foot-related functions in HV

In the HV group, the mean values (± SD) of five sections of FAOS were presented as follows: pain 70.05 ± 20.11, symptom 74.24 ± 20.99, Sports 76.17 ± 20.85, ADL 66.56 ± 20.06, QoL 51.55 ± 25.52, respectively.

Regarding the coronal components, only TMT joint rotation angle was significantly correlated with QoL (*p* < 0.01, *r* = − 0.716). As for the transverse deformities, neither IMA nor HVA significantly correlated with any foot-related function outcomes of FAOS. The details are demonstrated in Table 3.

**Table 3:** The relation between foot-related functions and deformities on different planes.

		Symptoms	Pain	ADL	Sports ability	QoL
Outcomes on the transverse plane	IMA	− 0.121	0.038	0.015	− 0.166	− 0.193
	HVA	− 0.291	− 0.271	− 0.335	− 0.474	− 0.383
Outcomes on the coronal plane	TMT joint rotation	0.001	− 0.390	− 0.473	− 0.449	− 0.716 **
	First MT torsion	0.295	0.193	0.249	0.325	0.332

*The correlation is significant with p-value < 0.05.

**The correlation is significant with p-value < 0.01.

## Discussion

This cross-observational comparative study aimed to determine the exact site of coronal deformity in HV and investigate whether the coronal deformity occurs independently of transverse-plane deformity. The results indicated that the rotation at the TMT joint might be the main contributor to the coronal deformity. The deformity on the coronal plane is independent of that on the transverse plane and is closely associated with foot function in HV patients.

### The site of coronal deformity

Since it is important to precisely regain normal alignment in multiplanar deformities, more and more studies have tried to clarify the coronal deformity in HV during the last decade. According to the CORA rationale in deformity correction^16^, determining the exact deformity site will guide the subsequent surgical procedure. The current study measured the TMT joint rotation and the bone torsion within the first MT for each subject by constructing a 3D model using high-resolution CT. We found a TMT joint rotation of 13.09 ± 4.29° in the HV group and 8.16 ± 2.05° in controls, with an average variation of 4.93°. This means the amount of TMT joint pronation in HV differs from that in the non-HV population. This result is consistent with the conclusion from several previous studies, which also found significant differences at TMT joint rotation^17–21^. Dayton et al.^21^ measured the ROM of the TMT joint during the operation and found 22.1° of supination was required to achieve normal alignment. Ornig et al. measured the angle between the external platform and the line at the MT base, which was parallel to the lateral cortex in weight-bearing conditions^22^. They found a significant difference between control and HV. The difference in the values between the current study and previous ones may be primarily attributed to the measurement method and definition of joint rotation. The view of the cutting plane is also crucial in angular measurement, and a standard and replicable procedure may be warranted in the future.

The different orientations of the TMT joint may be the result of joint instability, which has been mentioned in several studies^23–26^. The abnormal orientation of the TMT joint in HV feet may be due to repetitive joint motion across an extensive range. The surrounding structures fail to resist the pronation moment generated from weight-bearing. Besides, the position of the first ray partially relies on the agonist and antagonist muscles inserted into the first MT, i.e. peroneus longus (PL), tibialis anterior (TA) and tibialis posterior (TP). PL is a plantar flexor and evertor that inserts into the plantar side of the first MT base; cadaveric studies revealed that the PL plays a vital role in the first ray/medial column stability by reducing the TMT joint sagittal plane subluxation and intermetatarsal angle^27,28^. However, the metatarsal rotation is worsened simultaneously with the activation of the PL^28^. Further studies should investigate the relationship between the PL and TMT joint rotation in HV patients to clarify the effect of PL in HV development, especially deformities in the coronal plane.

As for the internal torsion of the first MT, we did not find a statistically significant abnormality in the HV subjects compared to the Control group. This contrasts with Cruz’s study^29^, whose control cohort of 45 patients (64 feet) presented with 3.45° torsion and Ota’s study^12^, which reported 4.7° torsion in their control cohort of 12 subjects (12 feet). This result shows that further investigation with larger sample sizes is warranted before a definitive conclusion regarding the presence of the first MT torsion can be drawn.

Up to now, multiple surgical procedures have been used to correct coronal deformity in Hallux valgus, including using MT osteotomies^30,31^ and arthrodesis at the TMT joint^32–34^. Precisely defining and correcting the deformity site should improve clinical outcomes and patient satisfaction. The current study’s result indicates that operating at the TMT joint may be the most promising outcome as it accurately tackles the coronal deformities at the CORA. Further studies are warranted to investigate the rotational correction power of TMT joint surgery, like the Lapidus procedure, by objectively analysing the post-operation coronal-plane anatomy.

Recently, Mahmoud et al.^35^ found that almost half of the subjects in the control group had an abnormal alpha angle greater than 16° (the value below the cut point should be regarded as a normal alpha angle according to Kim’s method^36^), indicating that the pronation may also exist in feet without HV. It should be noted that their coronal rotation may be affected by the pronation of the midfoot and hindfoot bones, as it used the ground level and the distal first MT as the measurement reference points. Our study isolated the medial cuneiform and the first MT to eliminate the influence of the more proximal hindfoot. Compared with plain radiographs, the current measurement can be more reliable in capturing multi-plane components of HV feet. A simple and reliable method may be required, which could be generalisable to clinical practice and help surgeons assess the specific deformity.

### The relation between coronal and transverse deformity in HV

The association between components on the coronal and transverse planes can further reveal the pathology of HV. The current study found that neither bone torsion angle nor TMT joint rotation angle was correlated with IMA or HVA, indicating that patients with large IMA may not experience joint rotation simultaneously. Likewise, patients with mild or moderate MT deviation may suffer obvious deformity on the coronal plane, which is, however, easily overlooked during routine radiological/physical examination. The result is consistent with the previous studies, which also investigated the correlation between the deformities on different planes^17,18,29,36^. As the extent of IMA and HVA on the plain radiographs does not predict the extent of coronal rotation, additional radiological/clinical documentation may be required.

HV patients in Campell’s study^18^ included juvenile and adult ones without subgroup analysis. Therefore, their results may be influenced by the spectrum of congenital HV, as they suspected that patients with congenital HV deformities had larger IMA and smaller pronation deformities compared to those with acquired HV. Our study recruited many middle-aged patients aged over 18. Further studies are required to investigate the possible factors predicting the pronation degree.

Our investigation into the association between deformity and foot-related function can further support the independence of coronal deformity. Similar to a previous study^7^, we found no significant correlation between foot function with the severity of transverse plane radiographic angles (IMA and HVA). Thordarson et al.^37^ found that variables on the plain radiographs (including IMA and HVA) did not influence patient perception. Mattews et al. also suggested that IMA and HVA did not fully explain a patient’s symptoms, as the HVA severity was not associated with the functional outcomes of the FAOS subscales^7^.

To our best knowledge, little evidence has clarified the relationship between coronal deformity degree and foot-related function. On the contrary, the joint rotation in our study was closely associated with QoL. The results demonstrated that a patient with a larger TMT joint rotation angle might experience worse QoL. The current results may partly explain that some patients, while only having mild IMA or HVA deformities, are extremely symptomatic with marked QoL reduction.

The information indicates that using the same surgery to treat this large heterogenous group of patients may not be the most ideal. Therefore, it may be necessary to examine the coronal anatomical alignment in addition to assessing the transverse plane deformity. Several studies raised a new classification regarding the severity of HV, which considered both transverse and coronal components^36,38^. Kim et al. found a group of patients who had abnormal first MT pronation without sesamoid deviation from the articular facet, suggesting a new category to assess HV deformity based on the MT pronation and sesamoid position using CT axial view^36^. Hatch et al. presented a triplane classification based on whether MT pronation, as well as a sesamoid subluxation, exist^38^. These novel systems to evaluate HV may help surgeons derive a more detailed and precise treatment plan. Although it met the sample size calculations, this study is too small to perform subgroup analysis. Therefore, further studies with a larger sample size are warranted for sub-group analysis to identify the possible categories of HV based on the first MT rotation and deviation. Besides, as the method in this study is novel, reliability and validity tests are also required in the future for better clinical application.

## Supplementary Information

Supplementary Information.

## Abbreviations

HV: Hallux valgus
MT: Metatarsal
TMT: Tarsometatarsal
MTP: Metatarsophalangeal
IMA: 1,2-Intermetatarsal angle
HVA: Hallux valgus angle
ROI: Range of interest

## References

[1] Nix S, Smith M, Vicenzino B (2010). Prevalence of hallux valgus in the general population: A systematic review and meta-analysis. J. Foot Ankle Res..

[2] Coughlin MJ, Jones CP (2007). Hallux valgus: Demographics, etiology, and radiographic assessment. Foot Ankle Int..

[3] Hurn SE, Matthews BG, Munteanu SE, Menz HB (2021). Effectiveness of nonsurgical interventions for hallux valgus: A systematic review and meta-analysis. Arthritis Care Res. (Hoboken).

[4] Heineman N, Liu G, Pacicco T, Dessouky R, Wukich DK, Chhabra A (2020). Clinical and imaging assessment and treatment of hallux valgus. Acta Radiol..

[5] Coughlin, M. J., Mann, R. A., Saltzman, C. L. *Surgery of the Foot and Ankle*. (Mosby St. Louis, 1999).

[6] Wagner, E., Wagner, P. Metatarsal pronation in hallux valgus deformity: A review. *J. Am. Acad. Orthop. Surg. Glob. Res. Rev.* **4** (2020). 10.5435/JAAOSGlobal-D-20-00091 PMC7322783 32656482

[7] Matthews M, Klein E, Youssef A, Weil L, Sorensen M, Weil LS, Fleischer A (2018). Correlation of radiographic measurements with patient-centered outcomes in hallux valgus surgery. Foot Ankle Int..

[8] Sierevelt IN, Zwiers R, Schats W, Haverkamp D, Terwee CB, Nolte PA, Kerkhoffs GMMJ (2018). Measurement properties of the most commonly used Foot- and Ankle-Specific Questionnaires: The FFI, FAOS and FAAM. A systematic review. Knee Surg Sports Traumatol. Arthrosc..

[9] Kanamoto S, Ogihara N, Nakatsukasa M (2011). Three-dimensional orientations of talar articular surfaces in humans and great apes. Primates.

[10] Matsuura Y, Ogihara N, Nakatsukasa M (2010). A method for quantifying articular surface morphology of metacarpals using quadric surface approximation. Int. J. Primatol..

[11] Kitashiro M, Ogihara N, Kokubo T, Matsumoto M, Nakamura M, Nagura T (2017). Age- and sex-associated morphological variations of metatarsal torsional patterns in humans. Clin. Anat..

[12] Ota T, Nagura T, Kokubo T, Kitashiro M, Ogihara N, Takeshima K, Seki H, Suda Y, Matsumoto M, Nakamura M (2017). Etiological factors in hallux valgus, a three-dimensional analysis of the first metatarsal. J. Foot Ankle Res..

[13] Drapeau MSM, Harmon EH (2013). Metatarsal torsion in monkeys, apes, humans and australopiths. J. Hum. Evol..

[14] Ito K, Tanaka Y, Takakura Y (2003). Degenerative osteoarthrosis of tarsometatarsal joints in hallux valgus: A radiographic study. J. Orthop. Sci. Off. J. Jpn. Orthop. Assoc..

[15] Coughlin MJ, Saltzman CL, Nunley JA (2002). Angular measurements in the evaluation of hallux valgus deformities: A report of the ad hoc committee of the american orthopædic foot & ankle society on angular measurements. Foot Ankle Int..

[16] Paley, D. (ed.) *Principles of Deformity Correction*. (Springer, 2005).

[17] Conti MS, Willett JF, Garfinkel JH, Miller MC, Costigliola SV, Elliott AJ, Conti SF, Ellis SJ (2020). Effect of the modified lapidus procedure on pronation of the first ray in hallux valgus. Foot Ankle Int..

[18] Campbell B, Miller MC, Williams L, Conti SF (2018). Pilot study of a 3-dimensional method for analysis of pronation of the first metatarsal of hallux valgus patients. Foot Ankle Int..

[19] Gómez Galván M, Constantino JA, Bernáldez MJ, Quiles M (2019). Hallux pronation in hallux valgus: Experimental and radiographic study. J. Foot Ankle Surg..

[20] Eustace S, O’Byrne J, Stack J, Stephens MM (1993). Radiographic features that enable assessment of first metatarsal rotation: The role of pronation in hallux valgus. Skelet. Radiol..

[21] Dayton P, Kauwe M, DiDomenico L, Feilmeier M, Reimer R (2016). Quantitative analysis of the degree of frontal rotation required to anatomically align the first metatarsal phalangeal joint during modified tarsal-metatarsal arthrodesis without capsular balancing. J. Foot Ankle Surg..

[22] Ornig, M., Tschauner, S., Holweg, P. L., Hohenberger. G. M., Bratschitsch, G., Leithner, A., Leitner, L. *A Novel Method of Clinical First Tarsometatarsal Joint Hypermobility Testing and Radiologic Verification*. (Wien Klin Wochenschr, 2020). 10.1007/s00508-020-01705-x PMC7969572 32617706

[23] Klaue K, Hansen ST, Masquelet AC (1994). Clinical, quantitative assessment of first tarsometatarsal mobility in the sagittal plane and its relation to hallux valgus deformity. Foot Ankle Int..

[24] Lee KT, Young K (2001). Measurement of first-ray mobility in normal vs. hallux valgus patients. Foot Ankle Int..

[25] Kimura T, Kubota M, Taguchi T, Suzuki N, Hattori A, Marumo K (2017). Evaluation of first-ray mobility in patients with hallux valgus using weight-bearing CT and a 3-D analysis system: A comparison with normal feet. J. Bone Jt. Surg. Am..

[26] Glasoe WM, Grebing BR, Beck S, Coughlin MJ, Saltzman CL (2005). A comparison of device measures of dorsal first ray mobility. Foot Ankle Int..

[27] Johnson CH, Christensen JC (1999). Biomechanics of the first ray part I. The effects of peroneus longus function: A three-dimensional kinematic study on a cadaver model. J. Foot Ankle Surg..

[28] Dullaert K, Hagen J, Klos K, Gueorguiev B, Lenz M, Richards RG, Simons P (2016). The influence of the Peroneus Longus muscle on the foot under axial loading: A CT evaluated dynamic cadaveric model study. Clin. Biomech. (Bristol, Avon).

[29] Cruz EP, Wagner FV, Henning C, Sanhudo JAV, Pagnussato F, Galia CR (2019). Does hallux valgus exhibit a deformity inherent to the first metatarsal bone?. J. Foot Ankle Surg..

[30] Dayton P, Carvalho S, Egdorf R, Dayton M (2020). Comparison of radiographic measurements before and after triplane tarsometatarsal arthrodesis for hallux valgus. J. Foot Ankle Surg..

[31] Dayton P, Feilmeier M, Kauwe M, Hirschi J (2013). Relationship of frontal plane rotation of first metatarsal to proximal articular set angle and hallux alignment in patients undergoing tarsometatarsal arthrodesis for hallux abducto valgus: A case series and critical review of the literature. J. Foot Ankle Surg..

[32] Lucijanic I, Bicanic G, Sonicki Z, Mirkovic M, Pecina M (2009). Treatment of hallux valgus with three-dimensional modification of mitchell’s osteotomy: Technique and results. J. Am. Podiatr. Med. Assoc..

[33] Şahin N, Cansabuncu G, Çevik N, Türker O, Özkaya G, Özkan Y (2018). A randomized comparison of the proximal crescentic osteotomy and rotational scarf osteotomy in the treatment of hallux valgus. Acta Orthop. Traumatol. Turc..

[34] Boychenko AV, Solomin LN, Belokrylova MS, Tyulkin EO, Davidov DV, Krutko DM (2019). Hallux valgus correction with rotational scarf combined with adductor hallucis tendon transposition. J. Foot Ankle Surg..

[35] Mahmoud K, Metikala S, Mehta SD, Fryhofer GW, Farber DC, Prat D (2020). The role of weightbearing computed tomography scan in hallux valgus. Foot Ankle Int..

[36] Kim Y, Kim JS, Young KW, Naraghi R, Cho HK, Lee SY (2015). A new measure of tibial sesamoid position in hallux valgus in relation to the coronal rotation of the first metatarsal in CT scans. Foot Ankle Int..

[37] Thordarson DB, Rudicel SA, Ebramzadeh E, Gill LH (2001). Outcome study of hallux valgus surgery—An AOFAS multi-center study. Foot Ankle Int..

[38] Hatch DJ, Santrock RD, Smith B, Dayton P, Weil L (2018). Triplane hallux abducto valgus classification. J. Foot Ankle Surg..

