# Supplementary material for: Investigation on the site of coronal deformities in Hallux valgus
Source: Sci Rep. 2023 Feb 1;13:1815. doi: 10.1038/s41598-023-28469-4 (PMC9892504; doi:10.1038/s41598-023-28469-4)

Supplementary information

Supplementary Fig 1: the scanning ROI in subjects with hallux valgus from the proximal phalanx to the navicular bone.


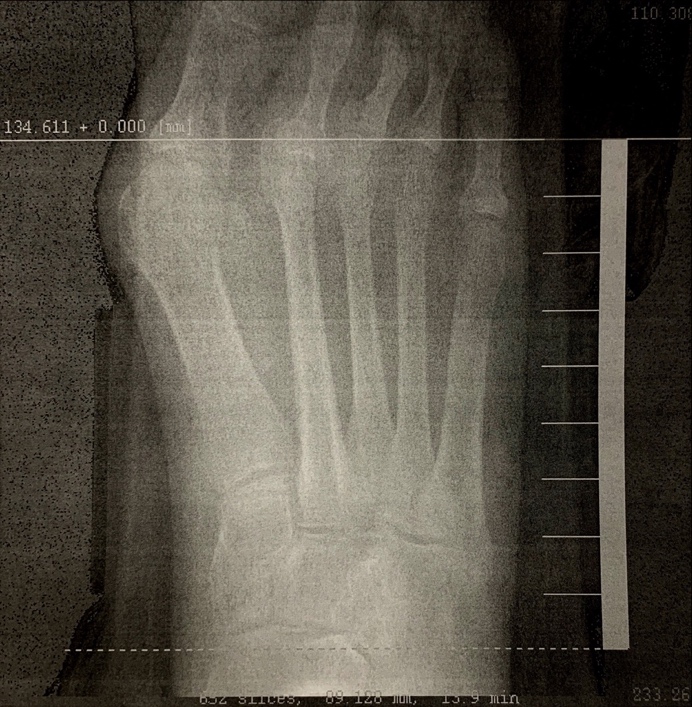

Supplement: Supplementary file 1 — Supplementary Information. [file 41598_2023_28469_MOESM1_ESM.docx]
